# Interactions between the volume effects of hydroxyethyl starch 130/0.4 and Ringer´s acetate

**DOI:** 10.1186/cc12749

**Published:** 2013-05-29

**Authors:** Robert G Hahn, Christian Bergek, Tobias Gebäck, Joachim Zdolsek

**Affiliations:** 1 Department of Anaesthesia, Faculty of Health Sciences, Linköping University, Garnisonsvägen, 58185 Linköping, Sweden; 2 Research Department, Södertälje Hospital, Rosenborgsgatan 6-10, 152 40 Södertälje, Sweden; 3 Department of Mathematical Sciences, Chalmers University of Technology, Maskingränd 2, 412 58 Gothenburg, Sweden. (The street addresses are really not needed for mail to reach any of these institutions

**Keywords:** pharmacokinetic model, i.v. fluids, hydroxyethyl starch

## Abstract

**Introduction:**

The turnover of Ringer´s solutions is greatly dependent on the physiological situation, such as the presence of dehydration or anaesthesia. The present study evaluates whether the kinetics is affected by previous infusion of colloid fluid.

**Methods:**

Ten male volunteers with a mean age of 22 years underwent three infusion experiments, on separate days and in random order. The experiments included 10 mL/kg of 6% hydroxyethyl starch 130/0.4 (Voluven™), 20 mL/kg of Ringer's acetate, and a combination of both, where Ringer´s was administered 75 minutes after the starch infusion ended. The kinetics of the volume expansion was analysed by non-linear least- squares regression, based on urinary excretion and serial measurement of blood haemoglobin concentration for up to 420 minutes.

**Results:**

The mean volume of distribution of the starch was 3.12 L which agreed well with the plasma volume (3.14 L) estimated by anthropometry. The volume expansion following the infusion of starch showed monoexponential elimination kinetics with a half-life of two hours. Two interaction effects were found when Ringer´s acetate was infused after the starch. First, there was a higher tendency for Ringer´s acetate to distribute to a peripheral compartment at the expense of the plasma volume expansion. The translocated amount of Ringer´s was 70% higher when HES had been infused earlier. Second, the elimination half-life of Ringer´s acetate was five times longer when administered after the starch (88 versus 497 minutes, *P *<0.02).

**Conclusions:**

Starch promoted peripheral accumulation of the later infused Ringer´s acetate solution and markedly prolonged the elimination half-life.

**Trial registration:**

ClinicalTrials.gov: NCT01195025

## Introduction

Intravenous infusion of acetated or lactated Ringer´s solution and hydroxyethyl starch (HES) are therapeutic cornerstones in modern perioperative fluid therapy. The turnover of such crystalloid fluids has been studied both in conscious [1,2] and anaesthetised [3] volunteers and in patients undergoing surgery [4,5]. Key findings include that the distribution half-life of 7 to 8 minutes is fairly constantly stable under most conditions, while the rate of elimination is retarded by 70 to 80% by general anaesthesia and surgery [6]. The slowing of the elimination by general anaesthesia is partly due to renin excretion [3], and therefore, can be alleviated by esmolol [7].

Less is known about the turnover of colloid fluid volumes and combinations of fluids. The half-life (*T*_1/2_) of HES is usually quantified based on the weight of the HES molecules in blood plasma. However, the smallest HES molecules are excreted, while the larger ones undergo sequential breakdown. The weight does not readily reflect the plasma volume expansion, as this is dependent on the number of molecules and not on their weight [8,9].

One clinical report suggests that administration of HES impairs plasma volume expansion and shortens the *T*_1/2 _of a subsequent infusion of Ringer´s acetate [10]. The present volunteer study explores this potential interaction in greater detail by comparing the volume kinetics of HES 130/0.4/9:1 (Voluven™) and acetated Ringer´s solution with a combination of both fluids. The hypothesis was that HES accelerates the distribution and/or the elimination of Ringer´s acetate.

## Methods

Ten male volunteers, aged 18 to 28 (mean, 22) years and with a body weight of 65 to 101 (mean, 79) kg, underwent three infusion experiments between August 2010 and February 2011. The study was approved by the regional ethics committee in Stockholm, Sweden (Dnr 2010/623-32) and registered at ClinicalTrials (NCT01195025). Each volunteer gave his consent for participation after being informed about the study both orally and in writing.

The experiments started between 7:30 and 8:30 am in the Department of Intensive Care at Linköping University Hospital. The volunteers had fasted since midnight, but they were allowed to eat one sandwich and drink one glass of liquid at 6 am. Upon arriving at the department, the volunteers were weighed on an electronic scale and then rested on a bed below an OPN Thermal Ceiling radiant warmer (Aragon Medical, River Vale, NJ, USA) placed about 1 m above them. The heat was adjusted to achieve maximum comfort. A cannula was placed in the cubital vein of each arm to infuse fluid and sample blood, respectively. Thirty minutes of rest to reach haemodynamic steady state was allowed before the experiments started.

### Infusions

Each volunteer underwent the following three experiments, in random order, separated by at least seven days.

**A**. HES, 10 mL/kg, over 30 minutes.

**B**. Ringer´s acetate, 20 mL/kg, over 30 minutes.

**C**. Combination of HES and Ringer's acetate; 10 mL/kg of HES was infused between 0 and 30 minutes, followed by 20 mL/kg of Ringer´s between 105 and 135 minutes.

The fluid volumes were provided according to the body weight obtained just before the study began. The first two volunteers in the Ringer´s-only group received only 15 mL/kg of fluid, due to uncertainty as to whether 20 mL/kg might cause excessive hypervolaemia in the combined experiment.

The colloid was hydroxyethyl starch 6% 130/0.4/9:1 (Voluven™, Fresenius Kabi, Bad Homburg, Germany; sodium 154 and chloride 154 mmol/L).

The crystalloid fluid was acetated Ringer´s solution (Baxter, Deerfield, IL, USA; sodium 130, chloride 110, acetate 30, potassium 4, calcium 2, and magnesium 1 mmol/L).

Both fluids were administered at room temperature (23°C) via infusion pumps (Volumat MC Agilia, Fresenius Kabi).

### Measurements

Venous blood (3 to 4 mL) was withdrawn using a vacuum tube from the venous cannula. A small volume of blood was drawn before each sampling, and the volume was replaced with 2 mL of 0.9% saline to prevent clotting. The venous blood was used to measure blood haemoglobin (Hb) concentration and haematocrit (Hct) on a Cell-Dyn Sapphire (Abbot Diagnostics, Abbot Park, IL, USA). Duplicate samples drawn at baseline, of which the mean was used, ensured a coefficient of variation of 1.2%.

The sampling intensity varied slightly, depending on the length of the experiment. In the Ringer´s experiment, blood was drawn every 5 minutes up to 60 minutes, and every 10 minutes thereafter up to 180 minutes. The same protocol was followed when starch alone was infused, but the follow-up continued with blood sampling every 30 minutes up to 420 minutes. In the combined experiment, the higher sampling intensity (every 5 minutes) was reinstituted for 60 minutes when the second infusion started.

Haemodynamic monitoring included non-invasive blood pressure, heart rate, and pulse oximetry. A comparison between Hb measured invasively and by pulse oximetry has been published elsewhere [11].

The volunteers voided upon entering the Department of Intensive Care, and this volume was discarded. They were allowed to void freely during the experiments, but remaining in the lying position. The total volume excreted was noted when they emptied their bladders at the end of the study.

### Kinetic calculations

**A**. The kinetics of HES was analysed using a one-volume fluid-space model. Fluid was then infused at a rate (*R*_o_) to increase the volume of the central body fluid space (*V*_c_) to a larger volume (*v*_c_). The rate of elimination (in mL/min) is given as the product of the volume expansion of *V*_c _and an elimination rate constant, (*k*_10_) (unit:min^-1^). *Perspiratio insensibilis *is accounted for by a zero-order clearance constant (*k*_o_), which was pre-set to 0.4 mL/min. The differential equation is:

$$\frac{\text{d}v_{\text{c}}}{\text{d}t}=R_{\text{o}}-k_{\text{o}}-k_{10}\left(v_{\text{c}}-V_{\text{c}}\right)$$

**B**. The kinetics of Ringer´s acetate was analysed using a two-volume fluid-space model, modified to allow for unbalanced distribution of fluid between *V*_c _and a peripheral compartment, (*V*_p_). Hence, distribution of fluid to *V*_p _is governed by a rate constant *k*_12_, and its return from *V*_p _to *V*_c _by another rate constant (*k*_21_) [7]. The differential equations are:

$$\begin{array}{l} \frac{\text{d}v_{\text{c}}}{\text{d}t}=R_{\text{o}}-k_{\text{o}}-k_{10}(v_{\text{c}}-V_{\text{c}})-k_{12}(v_{\text{c}}-V_{\text{c}})+k_{21}(v_{\text{p}}-V_{\text{p}})\\ \frac{\text{d}v_{\text{p}}}{\text{d}t}=k_{12}(v_{\text{c}}-V_{\text{c}})-k_{21}(v_{\text{p}}-V_{\text{p}}) \end{array}$$

When Ringer´s was infused, the elimination rate constant *k*_10 _was derived from the urinary excretion measured at the end of the each infusion experiment:

$$k_{10}=\frac{\sum \text{urine volume}}{AUC\;for\text{ (}v_{c}-V_{c}\text{)}}$$

**C**. The combined experiment was analysed using the one-volume model for HES and the two-volume model for acetated Ringer´s solution. Each curve-fit estimated five parameters. For HES, *k*_10 _was calculated based on the plasma dilution derived during the first 105 minutes. From 105 minutes onward, the measured plasma dilution was used to analyse the kinetics of both fluids; *k*_10 _for Ringer´s was then obtained as the difference between *k*_10 _for the combined experiment (see the equation above) and *k*_10 _for HES.

In all experiments, the Hb-derived fractional plasma dilution was used to indicate the volume expansion of *V*_c _resulting from the infusion:

$$\frac{v_{\text{c}}-V_{\text{c}}}{V_{\text{c}}}=\frac{\left[\text{Hb}/\text{hb}]-1\right)}{\left(1-\text{Hct}\right)}$$

where Hct is the haematocrit. Symbols in capital letters denote baseline values. A mathematical correction for the effect of blood sampling on the result was made [5].

The primary parameters in the models (*V*_c_, *k*_12_, and *k*_21_) were estimated by applying a non-linear least-squares regression routine (fminsearch) to the urinary excretion and the serial analyses of the fractional plasma dilution. The software used was Matlab R2010a (Math Works Inc., Natick, MA, USA).

The elimination half-life of the fluids (*T*_1/2_) was obtained as 0.693/*k*_10_.

A reference value for the expected size of the preoperative plasma volume was calculated individually, based on the weight (BW) and length (L) of the volunteers. The equation by Nadler *et al*. was used [12]:

$$\text{PV }\left(\text{L}\right)\;=\;\left[0.0\text{3219 BW}\left(\text{kg}\right)\;+\;0.\text{3669 }{\text{L}}^{3}\left(\text{m}\right)\;+\;0.\text{6}0\text{41}\right]\;\left(\text{1}-\text{haematocrit}\right).$$

Computer simulation of the volume expansion of *V*_c _and *V*_p _and the excreted fluid volume over time was performed by inserting the optimal parameter values for the group (Table 1) into the two differential equations describing the kinetic model, using the same software as the one used for the analyses. The accuracy of such simulations has been tested by comparing real data with the plasma dilution suggested by kinetic parameters derived from infusing fluid at various rates and volumes [6].

**Table 1:** Kinetic parameters.

	HES (*n *= 10)	Ringer's acetate (*n *= 10)	HES and Ringer's acetate* (*n *= 10)	Wilcoxon's matched-pair test
*V*_c _(L) for HES	3.07 (0.52)		3.18 (0.56)	*NS*
*V*_c _(L) for Ringer's		5.53 (4.25-7.31)	4.00 (3.05-4.82)	*P *< 0.03
*k*_12 _(10^-3 ^min^-1^)		20 (17-50)	41 (27-99)	*NS*
*k*_21 _(10^-3 ^min^-1^)		9 (3-27)	19 (10-27)	*NS*
*k*_10 _(10^-3 ^min^-1^) HES	6.5 (2.1)		6.4 (3.3)	*NS*
*k*_10 _(10^-3 ^min^-1^) Ringer's		7.3 (5.0-12.9)	1.7 (0.1-4.6)**	*P *< 0.03
*T*_1/2 _(min) HES	116 (35)		126 (75-150)	*NS*
*T*_1/2 _(min) Ringer's		88 (54-150)	497 (146-805)	*P *< 0.02

Data are the mean (SD) or median (25^th ^to 75th percentile) for the group. *The standard errors of the estimates (precision) were 4% for *V*_c _and *k*_12 _and 16% for *k*_21 _(median); **two negative values were set to the third lowest values in the series. HES, hydroxyethyl starch; *V*_c, _size of central body fluid space at baseline and during fluid therapy; *k*_12_, rate constant for fluid passing from *v*_c _to *v*_p_; *k*_21, _rate constant for fluid passing from *v*_p _to *v*_c_; *k*_10_, rate constant for fluid leaving the system; *T*_1/2_, half-life.

### Statistics

The optimal values of the kinetic parameters for each series of 10 infusions are reported as the mean (standard deviation) or, if distribution was skewed, as the median and 25th to 75th percentiles. Differences were evaluated by the Wilcoxon matched-pair test. Calculations were considered statistically significant if *P *<0.05.

## Results

### Kinetic analysis

The kinetic models were successfully fitted to all experiments.

**Figure 1:**
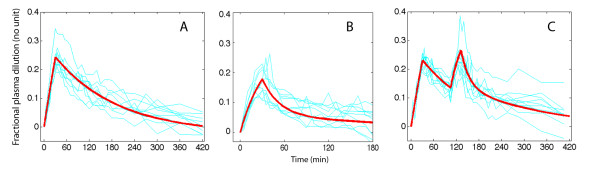
Measured plasma dilution in ten volunteers (thin lines) and modelled dilution using the optimal parameter estimates according to Table 1 (thick red lines), during and after intravenous infusion of **(A) **10 mL/kg of hydroxyethyl starch 130/0.4, **(B) **20 mL/kg of Ringer´s acetate, both over 30 minutes, and **(C) **a combination of both infusions where Ringer´s administration started after 105 minutes.

The plasma dilution over time in the three series of experiments is shown in Figure 1, and the optimal parameter estimates are shown in Table 1.

The two kinetic parameters for HES attained strikingly similar values in the two series of infusion experiments. *V*_c _was close to the plasma volume, which was 3.14 (0.25) L as estimated by anthropometry, and *T*_1/2 _was 2 hours.

*V*_c _for Ringer´s averaged 4.88 L, which was significantly larger than for HES (Wilcoxon´s test *P *<0.001). When combined with HES, distribution and elimination of Ringer´s occurred more slowly than in the single-infusion experiment (*P *<0.01).

The HES infusion induced diuresis while fluid was retained in the body after the combined experiment. The urinary excretion amounted to 85 (71 to 92)% of the infused volume during HES-alone but only to 53% (31 to 66)% during the combined experiment.

### Simulations

**Figure 2:**
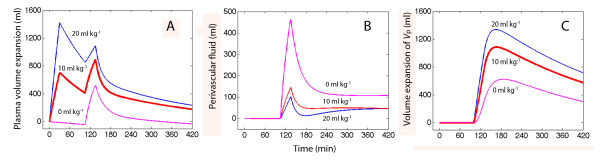
**Simulation of the expansion of the **(A) **plasma volume, **(B) **perivascular volume, and **(C) **in the peripheral body fluid space (*V*_p_) during the combined infusion experiment, where 20 mL/kg of Ringer´s acetate was infused between 105 and 135 minutes and preceded by no fluid (magenta), 10 mL/kg (red) or 20 mL/kg (blue) of hydroxyethyl starch 130/0.4**. The experiment generating the blue was not performed but simulated by computer using kinetic parameters for the 10 mL/kg dose.

The impact of infusing varying amounts of HES infused between 0 and 30 minutes on later distribution of Ringer´s acetate was explored by computer simulation (Figure 2). The simulations illustrated that the amount of infused HES greatly influences the distribution of a subsequent infusion of 20 mL/kg of Ringer´s solution. The greater the plasma volume expansion due to HES, the less effective Ringer´s became as a plasma volume expander (Figure 2A). Fluid residing in *V*_c _for Ringer´s, but outside *V*_c _for HES, which was considered to represent perivascular fluid, decreased when more HES was infused prior to Ringer´s (Figure 2B), while more fluid accumulated in the peripheral space, *V*_p _(Figure 2C).

**Figure 3:**
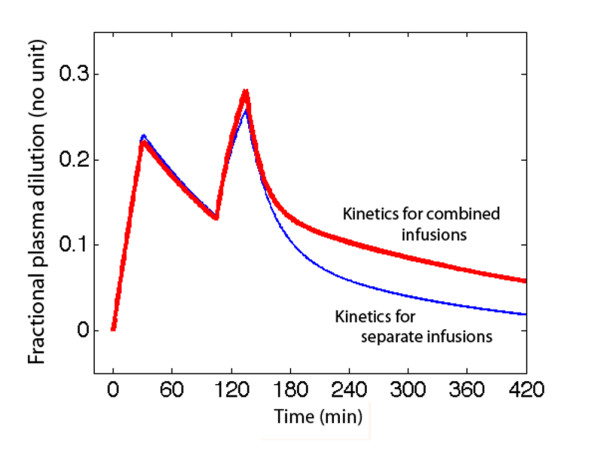
**Plasma dilution over time in the combined infusion experiment with computer simulation based on the optimal parameter estimates in Table 1 for the combined experiment (red) or the single-infusion experiments (blue)**.

The effect of the slower distribution and elimination of Ringer´s on the plasma dilution during the combined versus the single-infusion experiments is shown in Figure 3.

## Discussion

### Interaction effects

The study found two interaction effects when Ringer´s acetate was infused after HES. The first interaction effect was a higher tendency for Ringer´s to distribute to the peripheral compartment (*V*_p_) when preceded by HES. As shown in Figure 2C, the amount of Ringer´s in *V*_p _was 70% greater when 10 mL/kg of HES had been infused earlier. Rewriting this sub-plot by using uniform kinetic parameters reveals that 60% of the oedema-promoting effect of HES infusion is due to an assumption inherent in the kinetic model, namely, that distribution occurs faster when *V*_c _is already expanded. This is the case in the combined experiment, but not in the single-infusion experiment. The remainder of the increased oedema is due to differences in parameter estimates, primarily to the longer *T*_1/2 _when the infusion of Ringer´s acetate was preceded by HES.

The second interaction effect was a prolongation of the elimination *T*_1/2 _of Ringer´s. *T*_1/2 _was five times longer when administered after HES, compared to when Ringer´s acetate was infused alone (Table 1). The slow elimination could be due, in part, to the excess amount of chloride ions present in the HES preparation, which is based on normal saline. Renal clearance (urinary excretion/area under the curve (AUC) for plasma dilution) in the volunteers is 35 to 40% lower for normal saline than for lactated and acetated Ringer´s [1].

### Kinetics of HES and Ringer´s acetate

The kinetics of HES was well described by a one-volume model where expansion occurs solely within the plasma compartment. The kinetic parameters shown in Table 1 were derived from plasma data alone, as HES is not eliminated by urinary excretion, but governed by shifts in plasma colloid osmotic pressure (COP) caused by the enzymatic breakdown of HES molecules. The *T*_1/2 _of Voluven™ then averaged 116 minutes. Nevertheless, *T*_1/2 _did not differ much, being only 6 to 7 minutes shorter, when the urinary excretion was included in the curve-fitting procedure (calculations not shown).

Older data on the *T*_1/2 _for the plasma volume expansion from infusion of Voluven™ are difficult to find. The product monograph holds that the expansion lasts 4 to 6 hours [13]. The turnover of HES based on weight/volume measurements identifies a distribution *T*_1/2 _of 1.4 hours and elimination *T*_1/2 _of 12.1 hours [9]. Other studies yield elimination *T*_1/2 _of approximately 4 hours after infusing HES 130/0.40 [14] or 130/0.42 [15]. Although weight/volume measurements of HES is of interest in toxicology, such data cannot be used uncritically to infer plasma volume expansion over time [8].

The plasma volume kinetics of Ringer acetate is better known, but some new aspects were added, due to the use of a kinetic model with micro-constants. Least-squares regression was then based on volume changes rather than on plasma dilution, which increases the analysing power, as volume changes in *V*_p _can be calculated without knowledge of the size of *V*_p _(which can still be estimated in retrospect as *V*_c _*k*_12_/*k*_21_). Inclusion of the urinary excretion in the model even allowed analysis of potentially uneven distribution between *V*_c _and *V*_p_, which is of interest when studying interactions.

The results support that *V*_c _of Ringer´s acetate is larger than the plasma volume. The authors then assumed that some fluid quickly distributes to 'perivascular spaces' in highly perfused vascular beds (Figure 2B). In contrast, *V*_c _is close to the plasma volume, or even lower, during general anaesthesia [5,7]. Distribution has previously been reported to be completed 25 to 30 minutes post-infusion, which corresponds to a *T*_1/2 _of 7 to 8 minutes [6]. Here, allowing *k*_12 _and *k*_21 _to attain different values prolonged the time required until equilibrium was reached, at least for the singe-infusion experiment (Figure 3).

The elimination *T*_1/2 _of Ringer´s acetate was recently reported to average 21 minutes in well-hydrated, conscious volunteers, and 82 minutes in the presence of mild volume depletion [16]. The *T*_1/2 _of 88 minutes in the present single-infusion experiment suggests the mild volume depletion was present after the overnight fast despite ingestion of one glass of liquid 2 hours before the experiments started. The *T*_1/2 _of almost 500 minutes in the combined experiment differs greatly from is normally found in healthy volunteers. *T*_1/2 _of such magnitude has previously been reported only during laparoscopic surgery, which is characterised by fluid retention of multifactorial origin [4,7].

### Physiological correlates

Volume kinetics analyses fluid shifts between two functional body fluid spaces that may not correspond precisely to anatomical and physiological spaces. The key parameters *k*_12_, *k*_21 _and *k*_10 _are sufficient to describe the flow of infused fluid between these spaces (Figure 2) while the size of *V*_c _is needed only if the plasma dilution is simulated (Figures 1 and 3). The most rational view is still that *V*_c _corresponds to the plasma volume, although Ringer´s acetate rapidly fills also a small perivascular space, and that *V_p _*represents the interstitial fluid space.

Volume kinetics shows what happens with infused fluid in the body but does not outline the physiological background to the findings beyond the assumptions made in the kinetic model. Other methods must be incorporated to demonstrate reasons for the findings, such as the active modulation of the interstitial pressure by fibrocytes. However, most findings can be understood from the Starling equation. One example is that plasma volume expansion by starch promotes peripheral oedema when Ringer´s is infused later. Volume expansion from HES is maintained by a raised COP in the plasma compartment (*V*_c_). Ringer´s acetate can only increase the hydrostatic pressure and will therefore be translocated to peripheral tissues (*V*_p_) to maintain the balance of colloid and hydrostatic forces across the capillary wall.

The 'endothelial glycocalyx model' is a recently advocated theory that might serve as an adjunct to the Starling forces when modelling transcapillary fluid shifts [17]. Derived from microvascular research, this model holds that transcapillary leakage increases if the glycocalyx layer is damaged, which might occur from a number of stimuli including hypervolaemia-induced release of atrial natriuretic peptide [18]. In the present study, the strong linearity of the HES-alone elimination kinetics during 7 hours is inconsistent with any abrupt increase in vascular permeability. However, damage to the glycocalyx layer could explain the fraction of the increased peripheral translocation of Ringer´s that could not be predicted by letting the two single-infusion experiments predict the combined experiment.

The prolongation of the half-life of Ringer´s when provided together with HES might be due to inhibition of the filtration of water and solutes in the kidneys by means of a raised COP. This rise is possible due to the difference in excretion rate between the starch molecules (T_1/2 _approximately 12 hours) and the accompanying fluid volume (85% was excreted after 7 hours in this study).

### Clinical correlates

In clinical practice, HES and Ringer´s acetate are often combined. The described HES-induced peripheral accumulation of Ringer´s is likely to contribute to the tissue oedema frequently observed after lengthy surgery and intensive care. HES is also a part of 'goal-directed fluid therapy', which creates a controlled hypervolaemic state in open abdominal surgery and in debilitated patients undergoing acute surgery [19,20]. The use of HES is currently being questioned in septic [21] and intensive care [22] patients due a higher-than-expected need for post-infusion renal replacement therapy.

The effect of HES on the distribution of Ringer´s explains the findings by Borup *et al*. of minimal plasma volume expansion and high plasma clearance of lactated Ringer´s infused 4 hours after laparoscopic cholecystectomy [9]. Voluven™ had been administered in the absence of blood loss during the surgery, which must have caused hypervolaemia. The present study shows that, although distribution is accelerated, HES decreases rather than accelerates the elimination of later-infused crystalloid fluid.

### Limitations

A limitation of this study is that interactions were tested in volunteers and only in a hypervolaemic condition. Second, the infusions were given in a single sequence. Third, an alternative interpretation of the combined experiment is that the *T*_1/2 _for the decay of the HES-induced plasma volume expansion slowed down, instead of the reported prolongation of *T*_1/2 _for Ringer´s acetate. The number of study subject volunteers was on the low side. However, the explorative study design allowed us to provide all three study fluids to all volunteers. The consistent use of paired comparisons limited the influence of biological variability by always relying on paired comparisons.

## Conclusions

The plasma volume expansion following infusion of hydroxyethyl starch 130/0.4 (Voluven™) showed monoexponential elimination kinetics with an average half-life of 2 hours. The half-life of Ringer´s acetate solution was only slightly shorter, but the plasma volume decreased faster shortly after infusion due to extravascular distribution. Several changes of the turnover of Ringer´s acetate occurred when infused after Voluven™. These changes consisted in poorer plasma volume expansion, retarded elimination, and higher tendency to accumulate in peripheral tissues.

## Key messages

• The plasma volume expansion following infusion of hydroxyethyl starch 130/0.4 (Voluven™) had a half-life of approximately 2 hours.

• This half-life is much shorter than for weight/volume measurements of the starch.

• The half-life of Ringer´s acetate was only 24% shorter than for Voluven™.

• The tendency for Ringer´s acetate to cause peripheral oedema was increased when infused after starch.

• The elimination half-life of Ringer´s acetate was prolonged when preceded by starch.

## Abbreviations

AUC: area under the curve; COP: colloid osmotic pressure; Hb: haemoglobin; Hct: haematocrit; HES: hydroxyethyl starch; *k*_o_: fluid loss by evaporation through skin and airways; *k*_10_: rate constant for fluid leaving the system; *k*_12_: rate constant for fluid passing from *v*_c _to *v*_p_; *k*_21: _rate constant for fluid passing from *v*_p _to *v*_c_; R_o_: rate of infusion; *V*_c _and *v*_c: _size of central body fluid space at baseline and during fluid therapy: respectively; *V*_p _and *v*_p: _size of peripheral body fluid space at baseline and during fluid therapy: respectively; *T*_1/2_: half-life.

## Competing interests

Robert Hahn has provided paid lectures about fluid therapy for Baxter Healthcare. The other authors declare that they have no competing interests.

## Authors' contributions

RGH planned the study, made the calculations, and wrote the manuscript. CB conducted the infusion experiments. TG created the computer programs. JZ managed the logistics and organized the experiments. All authors read and approved the final manuscript.

## Authors' information

Robert G. Hahn, MD, PhD, is Research Director at Södertälje Hospital, Södertälje; Professor of Anaesthesia and Intensive Care at Linköping University; and Associate Professor at Karolinska Institutet, Stockholm, Sweden. Christian Bergek is a staff anaesthetist at Linköping University Hospital, Sweden. Tobias Gebäck is a PhD and Postdoctoral Fellow at the Department of Mathematical Sciences at Chalmers University of Technology, Gothenburg, Sweden. Joachim Zdolsek, MD, PhD, is senior anaesthetist at Linköping University Hospital, Sweden.
